# Overexpression of miR-4669 Enhances Tumor Aggressiveness and Generates an Immunosuppressive Tumor Microenvironment in Hepatocellular Carcinoma: Its Clinical Value as a Predictive Biomarker

**DOI:** 10.3390/ijms24097908

**Published:** 2023-04-26

**Authors:** Toshiaki Nakano, Chao-Long Chen, I-Hsuan Chen, Hui-Peng Tseng, Kuei-Chen Chiang, Chia-Yun Lai, Li-Wen Hsu, Shigeru Goto, Chih-Che Lin, Yu-Fan Cheng

**Affiliations:** 1 Graduate Institute of Clinical Medical Sciences, Chang Gung University College of Medicine, Taoyuan 333, Taiwan; sandy641014@hotmail.com (H.-P.T.); kueichenchiang@gmail.com (K.-C.C.); 2 Liver Transplantation Center, Department of Surgery, Kaohsiung Chang Gung Memorial Hospital, Kaohsiung 833, Taiwan; clchen@cgmh.org.tw (C.-L.C.); ann0401@cgmh.org.tw (I.-H.C.); hsuliwen1230@gmail.com (L.-W.H.); pochigoto0224@gmail.com (S.G.); immunologylin@gmail.com (C.-C.L.); 3 Liver Transplantation Center, Department of Diagnostic Radiology, Kaohsiung Chang Gung Memorial Hospital, Kaohsiung 833, Taiwan; may0313@cgmh.org.tw (C.-Y.L.); prof.chengyufan@gmail.com (Y.-F.C.); 4 Nobeoka Medical Check Center, Fukuoka Institution of Occupational Health, Nobeoka 882-0872, Japan

**Keywords:** hepatocellular carcinoma, exosomes, microRNAs, tumor cell migration, drug resistance, GAPDH, macrophage polarization, tumor microenvironment

## Abstract

Accumulating evidence suggests the involvement of tumor-derived exosomes in the development and recurrence of hepatocellular carcinoma (HCC). We previously identified miR-4669 as a highly expressed microRNA in circulating exosomes obtained from patients with post-transplant HCC recurrence. This study aimed to explore how overexpression of miR-4669 affects HCC development and recurrence. The impact of miR-4669 overexpression in Hep3B cells on tumor cell behavior and the tumor microenvironment was evaluated in vitro. In addition, the clinical value of exosomal miR-4669 for the prediction of treatment response to HCC downstaging therapies and following post-transplant HCC recurrence was explored. Overexpression of miR-4669 enhanced migration ability and led to acquired sorafenib resistance with an elevation of sirtuin 1 and long noncoding RNA associated with microvascular invasion. Active release of tumor-derived exosomes and glyceraldehyde 3-phosphate dehydrogenase (GAPDH) contributed to generating an immunosuppressive tumor microenvironment through the induction of M2 macrophage polarization. The retrospective analysis demonstrated the clinical value of exosomal miR-4669 for predicting treatment response to HCC downstaging therapies and for risk assessment of post-transplant HCC recurrence. In summary, the present data demonstrate the impact of exosomal miR-4669 on HCC recurrence through the enhancement of tumor aggressiveness and generation of an immunosuppressive tumor microenvironment.

## 1. Introduction

The liver is an essential organ for supporting many biological activities, including metabolism, digestion, detoxification, vitamin storage, and immunity [1]. It is also known as a silent organ, and there are often no symptoms, even when liver failure occurs. Primary liver cancer was the seventh most commonly diagnosed cancer and the third leading cause of cancer death worldwide in 2020 [2]. Hepatocellular carcinoma (HCC) is the most common form of primary liver cancer, and infection by the hepatitis B virus (HBV) and/or hepatitis C virus (HCV) is the main risk factor for HCC development [3]. Although HBV vaccination of newborns and the development of effective therapies for HBV and HCV have decreased rates of viral-associated HCC, the prevalence of metabolic risk factors for HCC, such as obesity, type II diabetes, and nonalcoholic fatty liver disease, may become the major cause of HCC globally [4]. HCC patients are often diagnosed at an intermediate to an advanced stage, which may reflect the higher mortality of the disease. Therefore, early detection of abnormal liver functions and subsequent treatment at an early stage are essential issues for maintaining liver homeostasis and promoting healthy aging.

There are several treatment regimens for HCC, including surgical resection, radiofrequency ablation, chemotherapy, locoregional therapy, proton therapy, and liver transplantation [5,6,7], and the choice of which depends on the tumor stage and condition of the patient, including economic capability. In general, the most effective treatment strategy for HCC is liver transplantation [8]. However, as mentioned above, HCC is often diagnosed at intermediate to advanced stages, and such patients are not suitable for liver transplantation; therefore, HCC downstaging should be performed to fit Milan or University of California at San Francisco (UCSF) criteria for candidates for liver transplantation [9,10]. The beneficial effect of downstaging therapy before liver transplantation has been reported in patients with defined profound tumor necrosis after treatment [11]. To evaluate treatment response to downstaging therapies, imaging assessments by computed tomography (CT) and magnetic resonance imaging (MRI) are generally performed. Nevertheless, the accuracy rate between imaging and pathological correlation was not satisfactory in our study [12]. In addition to imaging assessment, several biochemical indicators, such as alpha-fetoprotein (AFP), AFP lens culinaris agglutinin-3 (AFP-L3), and prothrombin induced by vitamin K absence-II (PIVKA-II, also known as des-γ-carboxyprothrombin, DCP), are clinically used for HCC surveillance [13], though the sensitivity and specificity of current biomarkers are relatively low. Therefore, the identification of reliable biomarkers for the evaluation of treatment response to downstaging therapies and prediction of post-transplant HCC recurrence is desirable for reducing HCC mortality as well as the cost of HCC therapies.

In the past decade, there have been many studies regarding the impact of tumor-associated extracellular vesicles, which contain microRNAs and long noncoding RNAs, on HCC development [14,15]. In our previous study, we demonstrated the impact of circulating exosomes on HCC development in an experimental animal model, and miR-92b was identified as a potential biomarker for the prediction of post-transplant HCC recurrence in a clinical setting [16]. Furthermore, our pilot study identified miR-4669 as one of the abundantly expressed microRNAs in circulating exosomes obtained from patients with post-transplant HCC recurrence [16]. To date, there is one paper regarding the expression profiles of circulating miR-4669 in colon cancer [17], yet there is no functional assessment of miR-4669 in HCC. In this study, we evaluated the impact of miR-4669 on tumor cell behavior and the tumor microenvironment. Additionally, the clinical value of circulating exosomal miR-4669 for prediction of treatment response to drug-eluting bead transarterial chemoembolization (DEB-TACE) and risk assessment of post-transplant HCC recurrence are discussed.

## 2. Results

### 2.1. Impact of miR-4669 Overexpression on Tumor Cell Growth and Migration in HCC

**Figure 1:**
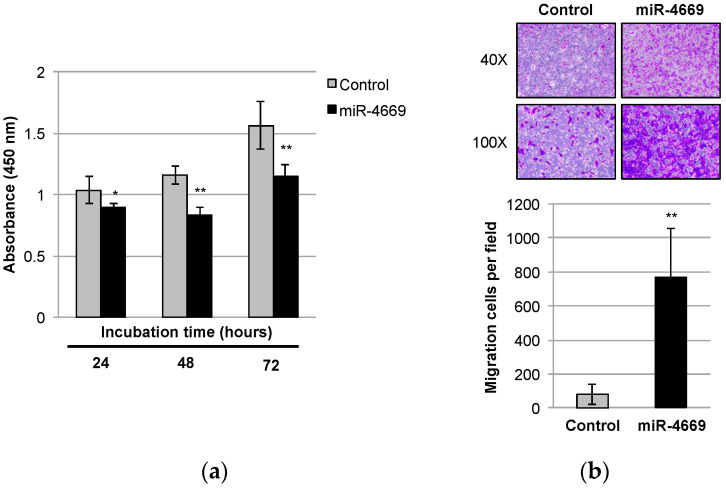
Impact of miR-4669 overexpression on tumor cell growth and migration in Hep3B cells. (**a**) After transfection with control or miR-4669 mimic (20 nM) for 24 h, Hep3B cells (5 × 10^3^ cells/well) were incubated for an additional indicated time. Cell viability was evaluated by using a cell proliferation assay. Data are represented as the mean ± standard deviation (SD) from three independent experiments. *, ** *p* < 0.05 and 0.01, respectively. (**b**) Control or miR-4669 mimic (20 nM)-transfected Hep3B cells (1 × 10^4^ cells/well) were incubated for 16 h, and cells that migrated to the Transwell membrane were counted using ImageJ software version 1.52p 22 June 2019 (National Institute of Health, Bethesda, MD, USA). Pictures are representative of three independent experiments. ** *p* < 0.01.

To explore the impact of miR-4669 overexpression on the malignant progression of HCC, the human HCC cell line Hep3B was transfected with miR-4669 mimic (20 nM) or control mimic (20 nM) for 24 h, followed by incubation for 24, 48, and 72 h. As shown in Figure 1a, miR-4669 overexpression affected the growth ability of Hep3B cells compared with the control. On the other hand, miR-4669 overexpression significantly enhanced the migration of Hep3B cells (Figure 1b). These results suggest the impact of miR-4669 on malignant phenotype development rather than on tumor initiation and maintenance.

### 2.2. Impact of miR-4669 Overexpression on Drug Sensitivity in HCC

**Figure 2:**
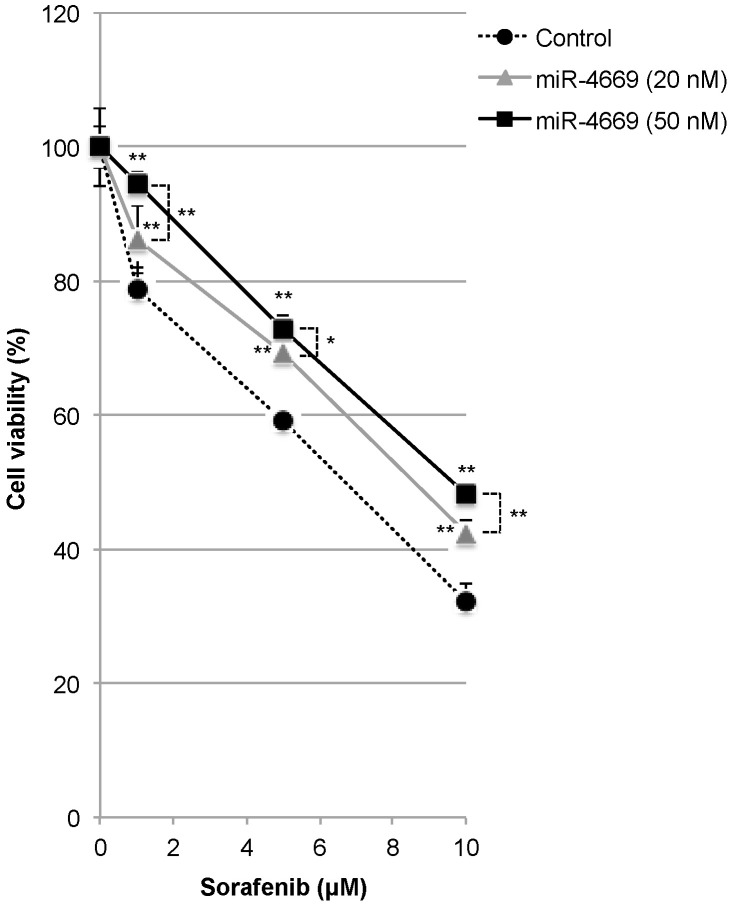
Impact of miR-4669 overexpression on drug resistance in Hep3B cells. Control (50 nM) or miR-4669 mimic (20 or 50 nM)-transfected Hep3B cells (1 × 10^4^ cells/well) were treated with sorafenib (1, 5, and 10 μM) for 24 h, and cell viability was evaluated. Hep3B cells were treated with 10% DMSO as a control for drug-induced cytotoxicity. Data are representative of three independent experiments. *, ** *p* <0.05 and 0.01, respectively.

Induction of drug resistance is one of the hallmarks of tumor aggressiveness in HCC [18]. We, therefore, explored the impact of miR-4669 overexpression on drug sensitivity. As shown in Figure 2, overexpression of miR-4669 led to the acquisition of sorafenib resistance in a transfection dose-dependent manner.

### 2.3. Impact of miR-4669 Overexpression on Genes Involved in Metastasis and Drug Resistance

**Figure 3:**
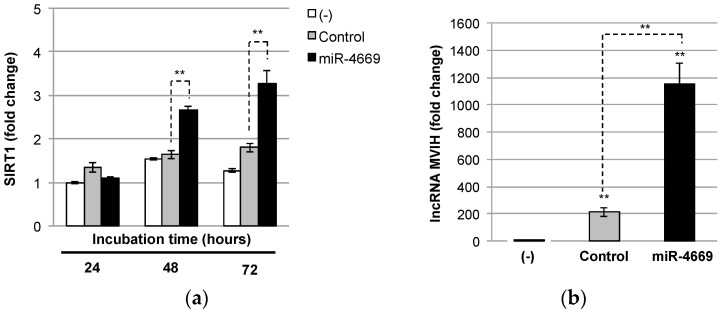
Impact of miR-4669 overexpression on expression levels of SIRT1 and lncRNA MVIH. (**a**) Intact (-), control, or miR-4669 mimic (20 nM)-transfected Hep3B cells were incubated for 24, 48, and 72 h, and SIRT1 levels were measured by quantitative real-time PCR. Data are represented as the mean ± SD from three independent experiments. ** *p* < 0.01. (**b**) Intact (-), control, or miR-4669 mimic (20 nM)-transfected Hep3B cells were incubated for 48 h, and the lncRNA MVIH level was measured by quantitative real-time PCR. Data are represented as the mean ± SD from three independent experiments. ** *p*< 0.01.

There are several studies regarding the impact of sirtuin 1 (SIRT1) on promoting metastasis and drug resistance [19,20,21]. Microvascular invasion (MVI) is a crucial risk factor associated with metastasis and postoperative recurrence of HCC [22,23], and long noncoding RNA associated with MVI in HCC (lncRNA MVIH) acts as a predictor of poor prognosis after hepatectomy [24].We hence explored the impact of miR-4669 overexpression on expression levels of SIRT1 and lncRNA MVIH. As shown in Figure 3a, there was no obvious change in SIRT1 expression in intact or control mimic-transfected Hep3B cells, but miR-4669 overexpression gradually increased SIRT1 expression in a time-dependent manner. Furthermore, a significant elevation of lncRNA MVIH was observed in miR-4669-transfected Hep3B cells (Figure 3b).

### 2.4. Impact of Tumor-Derived Extracellular Factors on Macrophages in the Tumor Microenvironment

**Figure 4:**
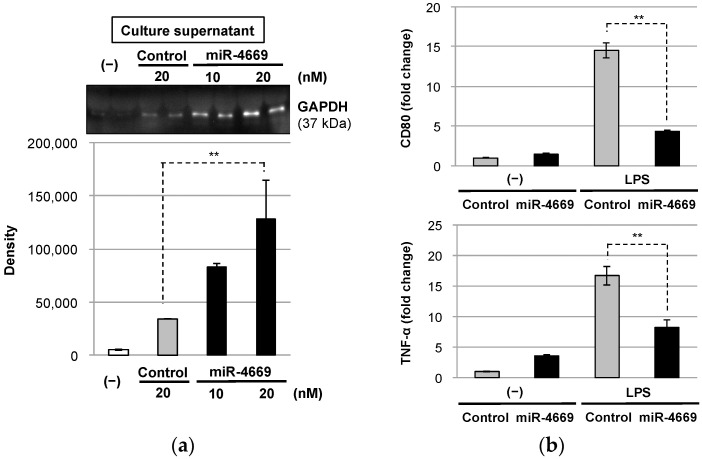
Impact of tumor-derived extracellular factors on macrophages in the tumor microenvironment. (**a**) Control (20 nM) or miR-4669 mimic (10 or 20 nM)-transfected Hep3B cells (1 × 10^4^ cells/well) were harvested, and GAPDH levels in the culture medium (10 μL) were semi-quantified using western blot analysis. The mean total protein concentration of the culture medium was comparable to the two groups (control: 4.23 mg/mL, miR-4669: 4.06 mg/mL). ** *p* < 0.01. (**b**) RAW264.7 cells were incubated with tumor-derived exosomes obtained from the culture medium of control or miR-4669mimic (20 nM)-transfected Hep3B cells for 24 h with or without lipopolysaccharide (LPS, 1 μg/mL) stimulation. Expression levels of CD80 and TNF-α were then measured by quantitative real-time PCR. Data are represented as the mean ± SD from three independent experiments. ** *p* < 0.01.

The generation of an immunosuppressive tumor microenvironment, which leads to evasion of the host’s immune surveillance, is one of the hallmarks of aggressive tumor cells [25]. In our previous study, we demonstrated the involvement of glyceraldehyde 3-phosphate dehydrogenase (GAPDH) release in terminating M1 macrophage-mediated proinflammatory responses by inducing M2 macrophage polarization [26]. GAPDH is one of the enzymes associated with glycolysis, and overexpression of GAPDH is associated with tumor development [27,28]. We speculate that the malignant phenotype of tumor cells actively releases GAPDH to generate an immunosuppressive tumor microenvironment and explored the impact of miR-4669 overexpression on the extracellular release of GAPDH by Hep3B cells. As shown in Figure 4a, Hep3B cells overexpressing miR-4669 actively released GAPDH, suggesting an indirect impact of miR-4669 overexpression in Hep3B cells on M2 macrophage polarization through the induction of extracellular GAPDH release.

As shown in supplementary Figure S1, Hep3B cells overexpressing miR-4669 extracellularly released miR-4669-enriched exosomes. To further explore the impact of tumor-derived exosomes on macrophage polarization, murine macrophage cell line RAW264.7 cells were incubated with tumor-derived exosomes purified from the culture media of control or miR-4669 mimic-transfected Hep3B cells. As depicted in Figure 4b, miR-4669-enriched exosomes suppressed CD80 and TNF-α in RAW264.7 cells, suggesting a direct impact of exosomal miR-4669 on the suppression of M1 macrophage activity to generate an immunosuppressive tumor microenvironment.

### 2.5. Clinical Value of Exosomal miR-4669 for Prediction of Treatment Response to HCC Downstaging Therapies and for Risk Assessment of Posttransplant HCC Recurrence

In a retrospective cohort study, we enrolled 47 HCC patients who received HCC downstaging therapies, including conventional TACE and DEB-TACE, as candidates for living donor liver transplantation (LDLT) (Table 1). These patients were divided into two groups: the HCC no recurrence group for more than 5 years (*n* = 16) and the HCC recurrence group within 2 years after LDLT (*n* = 31).

**Figure 5:**
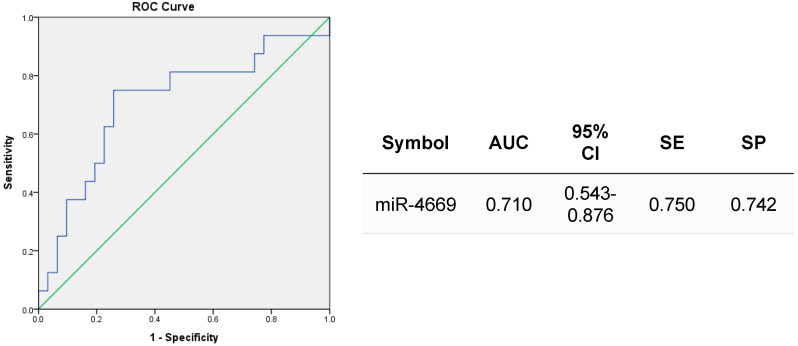
Clinical verification of exosomal miR-4669 expression. A ROC curve revealed theclinical value of pre-transplant exosomal miR-4669 levels for the prediction of post-transplant HCC recurrence. AUC: area under the curve, CI: confidence interval, SE: sensitivity, SP: specificity.

A receiver operating characteristic (ROC) curve was plotted according to the expression profile of circulating exosomal miR-4669 after HCC downstaging (before LDLT) in each patient (Figure 5). The data revealed a fair predictive accuracy of pre-transplant exosomal miR-4669 levels for risk assessment of post-transplant early HCC recurrence (*p* = 0.020). The cutoff value was a 13.1-fold change compared with non-HCC patients (negative control) [16].

As a validation cohort study, we enrolled 43 HCC patients and further explored the impact of exosomal miR-4669 on treatment response to DEB-TACE with different particle sizes (DC-Bead^TM^: 100–300 μm, n = 23, Tandem^TM^: 100 μm, n = 17, HepaSphere^TM^: 30–60 μm, n = 3) and following post-transplant HCC recurrence. As illustrated in Table 2, ninepatients (39.1%) had experienced HCC recurrence after LDLT, and most patients with HCC recurrence (7/9, 77.8%) revealed higher levels of exosomal miR-4669 after DC-Bead^TM^ TACE for downstaging.

On the other hand, four patients (23.5%) experienced HCC recurrence after LDLT, with higher levels of exosomal miR-4669 after Tandem^TM^ TACE for downstaging (Table 3). Although the case number was quite small, no significant elevation of exosomal miR-4669 was found in patients with HepaSphere^TM^ TACE for downstaging, and they had a good prognosis after LDLT (Table 4).

Taken together, the present data suggest the clinical value of pre-transplant exosomal miR-4669 levels for predicting treatment response to DEB-TACE and for risk assessment of post-transplant HCC recurrence.

## 3. Discussion

Although the involvement of tumor-derived extracellular vesicles in HCC progression and recurrence has been well recognized [14,15], the mode of action of exosomal components in HCC is not fully understood. In this study, we demonstrate the impact of circulating exosomal miR-4669, a highly expressed microRNA in patients with post-transplant HCC recurrence [16], on tumor cell biology (Figure 1, Figure 2 and Figure 3) and the tumor microenvironment (Figure 4). In addition, the clinical value of circulating exosomal miR-4669 as a potential biomarker for the prediction of treatment response to DEB-TACE and risk assessment of post-transplant HCC recurrence is discussed (Figure 5, Table 2, Table 3 and Table 4).

Specifically, we demonstrate that miR-4669 overexpression in Hep3B cells enhances their migration ability but suppresses tumor cell growth (Figure 1). Reduced proliferation and enhanced migration are hallmarks of the development of malignant phenotypes [29]. MiR-4669 overexpression is also associated with sorafenib resistance (Figure 2), and elevation of SIRT1 and lncRNA MVIH by miR-4669 overexpression (Figure 3) may play an important role in the development of a malignant phenotype. Although no study has demonstrated direct crosstalk between SIRT1 and lncRNA MVIH, SIRT1 overexpression in human HCC specimens is associated with microvascular invasion [30].There are also several studies regarding the therapeutic potential of SIRT1 inhibition in HCC [31,32]. Further studies are necessary to evaluate the therapeutic potential of miR-4669 inhibitors in HCC.

Interestingly, miR-4669 overexpression not only affects tumor cell biology but also modulates the tumor microenvironment (Figure 4). Tumor-associated macrophages (TAMs) are known to play an important role in HCC progression [33], and active secretion of GAPDH by miR-4669 overexpression in Hep3B cells may generate an immunosuppressive tumor microenvironment through M2 macrophage polarization [26]. Indeed, tumor cells prefer to use aerobic glycolysis for adenosine triphosphate (ATP) production [34], and molecular mechanisms of glycolysis and lactate (the end product of aerobic glycolysis) for M2 macrophage polarization have recently been proposed [35]. Despite no direct evidence to support the impact of miR-4669 on M2 macrophage polarization, downregulation of circulating exosomal miR-4669 is reported as a novel biomarker for early prediction of effective treatment response to subcutaneous immunotherapy (SCIT) in pediatric patients with allergic rhinitis [36], which is associated with type 2 immune responses, and M2 macrophages correlate with disease severity [37,38]. Further studies are necessary to illustrate the mode of action of miR-4669 and its inhibitor on aerobic glycolysis and TAMs for generating an immunosuppressive tumor microenvironment in HCC.

In addition to the effect of miR-4669 overexpression in Hep3B cells, we herein demonstrate the clinical value of circulating exosomal miR-4669 levels for the prediction of treatment response to DEB-TACE and risk assessment of post-transplant HCC recurrence (Figure 5, Table 2, Table 3 and Table 4). Indeed, based on imaging assessment, all HCC patients in the retrospective and validation cohorts experienced partial to complete response to DEB-TACE and were judged as having successful HCC downstaging suitable for LDLT. However, 30.2% (13/43) of the HCC patients with DEB-TACE in a validation cohort experienced post-transplant HCC recurrence, and 84.6% (11/13) had higher levels of exosomal miR-4669 before LDLT (Table 2, Table 3 and Table 4). Therefore, reduced expression of exosomal miR-4669 after DEB-TACE may be a key indicator in the assessment of successful HCC downstaging with a reduced risk of post-transplant HCC recurrence. In our previous study, we demonstrated the impact of particle size (100–300 μm vs. 300–500 μm) on the safety and efficacy of DAB-TACE [39]. A similar observation has been reported by another group [40]. In this study, we found that the use of smaller particle sizes (≤100 μm) of DAB might further reduce the risk of post-transplant HCC recurrence [>100 μm: 39.1% (9/23) vs. ≤100 μm: 20.0% (4/20)]. As support for our observation, a recent study highlighted the safety and efficacy of DAB-TACE with a smaller particle size (<100 μm) [41]. On the other hand, Zhang J et al. reported a better safety profile of medium-size (300–500 μm) microspheres than small microspheres (100–300 μm) for DAB-TACE [42]. Further studies are necessary to demonstrate the impact of particle size on the efficacy and safety of DAB-TACE as well as impacts on the expression profile of circulating exosomal miR-4669 during HCC downstaging.

In summary, the present data show the impact of circulating exosomal miR-4669 on post-transplant HCC recurrence through the enhancement of tumor aggressiveness and generation of an immunosuppressive tumor microenvironment. Overall, evaluation of circulating exosomal miR-4669 before/after DAB-TACE or LDLT may be effective for predicting treatment response to HCC downstaging therapies and for risk assessment of post-transplant HCC recurrence. Further large-scale analysis with multiple medical centers should be conducted to evaluate whether exosomal miR-4669 is an effective biomarker for the prediction of treatment response to DAB-TACE and subsequent outcomes after LDLT.

## 4. Materials and Methods

### 4.1. Cell Culture and Treatment

Hep3B cells were obtained from the Bioresource Collection and Research Center (Hsinchu, Taiwan). To explore the impact of miR-4669 overexpression on cell viability and migration, Hep3B cells were transfected with a miR-4669 mimic (10, 20, or 50 nM) or a negative control mimic (Qiagen, Hilden, Germany) according to a previous study [16]. After transfection for 24 h, cell viability was evaluated using a Cell Counting Kit-8 (CCK-8) assay (MilliporeSigma, Burlington, MA, USA). Migration assays were performed according to a previous study [16], and migrated cells at the bottom of the Transwell membrane (8 μm pore size, MilliporeSigma) were stained with 0.2% crystal violet for cell counting.

### 4.2. Drug Sensitivity Assay

To explore the impact of miR-4669 overexpression on drug sensitivity, control (50 nM) or miR-4669 mimic (20 or 50 nM)-transfected Hep3B cells were treated with sorafenib (1, 5, and 10 μM) for 24 h. Cell viability was evaluated using a CCK-8 assay and calculated by the following formula:

Cell viability (%) = 100 × (Absorbance of sorafenib-treated Hep3B cells − Absorbance of 10% DMSO-treated Hep3B cells)/(Absorbance of intact Hep3B cells − Absorbance of 10% DMSO-treated Hep3B cells)

### 4.3. RNA Extraction and Quantitative Real-Time PCR

Total RNA (3 μg) was extracted from cell lines using TRIzol (Thermo Fisher Scientific, Waltham, MA, USA) and reverse-transcribed using a High Capacity Reverse Transcription Kit (Thermo Fisher Scientific). Expression levels of SIRT1 and lncRNA MVIH were quantified using quantitative real-time PCR (Applied Biosystems, Waltham, MA, USA) with the specific primer sets shown in Table 5. β-Actin was used as an internal control for normalization.

### 4.4. Western Blot Analysis

Control (20 nM) or miR-4669 mimic (10 or 20 nM)-transfected Hep3B cells were cultured for 72h, and GAPDH levels in the culture medium were semi-quantified using western blot analysis. Briefly, proteins in culture media (10 μL) were fractionated and transferred onto a PVDF membrane (GE Healthcare Bio-Sciences Corp., Piscataway, NJ, USA). The membrane was immunoprobed with a rabbit anti-GAPDH monoclonal antibody (×500 dilution, Abcam, Cambridge, UK) followed by incubation with HRP-conjugated anti-rabbit IgG (×5000 dilution, Jackson ImmunoResearch Laboratories, Inc., West Grove, PA, USA). Signals were visualized using an Immunobilon Forte Western HRP substrate (MilliporeSigma), and signal intensity was quantified using a G:BOX Image Station iChemi XL device (SYNGENE, Cambridge, UK).

### 4.5. Tumor-Derived Exosome Isolation and Macrophage Polarization

Control or miR-4669 mimic (20 nM)-transfected Hep3B cells were cultured for 24 h in a culture medium supplemented with exosome-depleted FBS (System Biosciences, Mountain View, CA, USA). Tumor-derived exosomes were purified from 10 mL of the culture supernatant by using ExoQuick-TC Exosome Precipitation Solution (System Biosciences). Purified exosomes were dissolved in the culture medium, and RAW264.7 cells (Bioresource Collection and Research Center) were cultured for 24 h under LPS (1 μg/mL) stimulation. Expression levels of M1 macrophage markers (CD80 and TNF-α) in RAW264.7 cells were quantified using quantitative real-time PCR (Applied Biosystems) with the specific primer sets shown in Table 6.

### 4.6. Clinical Verification

Exosomal RNAs were purified from 250 μL of serum by using an ExoQuick Exosome Precipitation Solution (System Biosciences) and a SeraMir serum/plasma Exosome RNA Purification Kit (System Biosciences). MiR-39 from *Caenorhabditis elegans* was used as an internal control for normalization according to the manufacturer’s recommendation. The expression signature of exosomal miR-4669 was indicated as a fold-change compared with the value of non-HCC patients (negative control) according to a previous study [16].

### 4.7. Statistical Analysis

Unpaired two-tailed Student’s *t*-test was used to determine the significance of the difference between normally distributed means of values in two groups. Each sample was tested in triplicate, and results are indicated as the mean ± standard deviation (SD). For clinical verification, the Mann—Whitney U test was applied. Statistical analysis was performed using SPSS software version 22 (IBM, Armonk, NY, USA) and GraphPad Prism software version 7 (GraphPad Software, La Jolla, CA, USA). A value of *p* < 0.05 was considered to be significant.

## Figures and Tables

**Table 1:** Patient demographics after HCC downstaging (before LDLT).

	HCC No Recurrence (>5 Years)	HCC Recurrence (≤2 Years)
Gender (female/male)	0/16	4/27
Age (years)	51.4 (31.2–66.1)	53.6 (26.3–65.1)
AST (U/L)	40.8 (16–83)	64.4 (26–143)
ALT (U/L)	39.9 (10–79)	48.2 (13–162)
AFP (ng/mL)	267.9 (3–4385)	76.9 (3–410.8)
Tumor number	1.69 (0–6)	3.17 (1–11)
Tumor total size (cm)	3.26 (0–8)	8.16 (2.7–20.3) **

AST: aspartate transaminase, ALT: alaninetransaminase, AFP: α-fetoprotein, ** *p* < 0.01.

**Table 2:** HCC patients with DC-Bead^TM^ TACE for downstaging.

Patient	Pathology	HCC Recurrence	Exosomal miR-4669 (Fold Change)
DC-1	CR	No	6.5
DC-2	CR	No	3.3
DC-3	PR	Late	**31.1**
DC-4	PR	Early	6.3
DC-5	PR	Early	12.3
DC-6	PR	Early	**59.0**
DC-7	PR	Early	**41.4**
DC-8	PR	No	7.1
DC-9	PR	No	3.1
DC-10	PR	Early	**25.8**
DC-11	PR	No	9.3
DC-12	PR	No	5.0
DC-13	PR	No	2.4
DC-14	PR	No	5.9
DC-15	PR	Early	**37.3**
DC-16	PR	No	4.5
DC-17	PR	No	4.4
DC-18	PR	No	7.2
DC-19	PR	Late	**27.3**
DC-20	PR	No	2.2
DC-21	PR	No	4.5
DC-22	PR	No	4.7
DC-23	PR	Early	**40.0**

CR: complete response, PR: partial response, CCC: cholangiocarcinoma, Early: HCC recurrence within 2 years after LDLT, Late: HCC recurrence at >2 years after LDLT, Bold red: over the cut-off value (13.1-fold change).

**Table 3:** HCC patients with Tandem^TM^ TACE for downstaging.

Patient	Pathology	HCC Recurrence	Exosomal miR-4669 (Fold Change)
Tan-1	CR	Early	**25.8**
Tan-2	PR	Early	**13.1**
Tan-3	PR	No	5.7
Tan-4	PR	No	2.4
Tan-5	PR	Early	**21.0**
Tan-6	PR	No	2.0
Tan-7	PR	No	4.3
Tan-8	PR	No	1.4
Tan-9	PR	No	5.0
Tan-10	PR	No	1.6
Tan-11	PR	No	2.0
Tan-12	PR	No	1.9
Tan-13	PR	No	9.0
Tan-14	PR	No	1.8
Tan-15	PR	No	7.4
Tan-16	CCC	Early	**24.9**
Tan-17	CCC	No	9.8

CR: complete response, PR: partial response, CCC: cholangiocarcinoma, Early: HCC recurrence within 2 years after LDLT, Bold red: over the cut-off value (13.1-fold change).

**Table 4:** HCC patients with HepaSphere^TM^ TACE for downstaging.

Patient	Pathology	HCC Recurrence	Exosomal miR-4669 (Fold Change)
Hep-1	CR	No	4.6
Hep-2	PR	No	7.2
Hep-3	PR	No	9.0

CR: complete response, PR: partial response.

**Table 5:** Primer information (for Hep3B cells).

	Forward Primer	Reverse Primer
SIRT1	5′-TAGACACGCTGGAACAGGTTGC-3′	5′-CTCCTCGTACAGCTTCACAGTC-3′
lncRNA MVIH	5′-AATTTTGCACATCTGAACAGCC-3′	5′-TTCAAAATCCCACTACGCCCA-3′
β-actin	5′-CACCATTGGCAATGAGCGGTTC-3′	5′-AGGTCTTTGCGGATGTCCACGT-3′

**Table 6:** Primer information (for RAW264.7 cells).

	Forward Primer	Reverse Primer
CD80	5′-GCAGGATACACCACTCCTCAA-3′	5′-AAAGACGAATCAGCAGCACAA-3′
TNF-α	5′-ATGAGCACAGAAAGCATGATC-3′	5′-TACAGGCTTGTCACTCGAATT-3′
β-actin	5′-GGCTGTATTCCCCTCCATCG-3′	5′-CCAGTTGGTAACAATGCCATGT-3′

## Data Availability

No datasets were analyzed or generated during the study.

## Supplementary Materials

The following supporting information can be downloaded at: https://www.mdpi.com/article/10.3390/ijms24097908/s1.
