# Supplementary material for: Overexpression of miR-4669 Enhances Tumor Aggressiveness and Generates an Immunosuppressive Tumor Microenvironment in Hepatocellular Carcinoma: Its Clinical Value as a Predictive Biomarker
Source: Int J Mol Sci. 2023 Apr 26;24(9):7908. doi: 10.3390/ijms24097908 (PMC10177802; doi:10.3390/ijms24097908)
Supplement: Supplementary file 1 [file ijms-24-07908-s001.zip › ijms-2316375-supplementary.pdf]

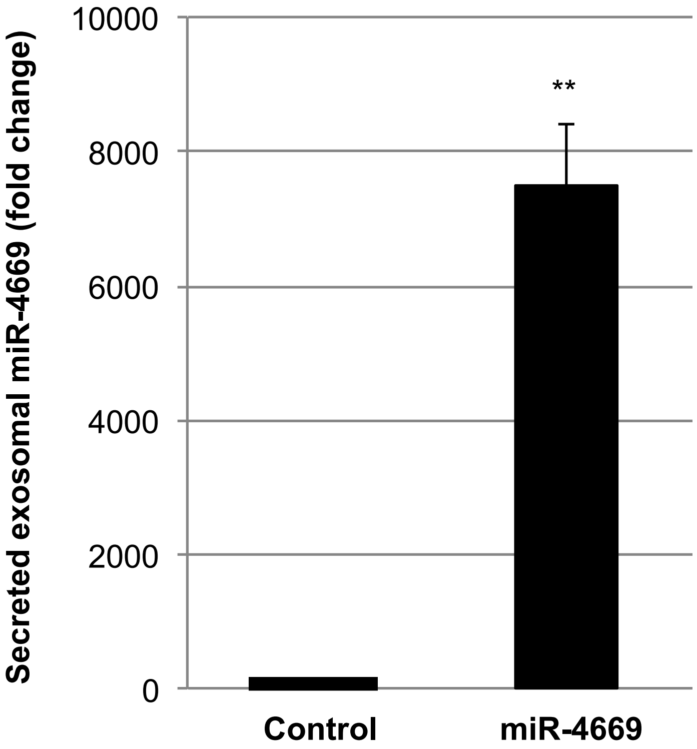

Figure S1. Extracellular release of exosomal miR-4669 in Hep3B cells overexpressing miR-4669. Hep3B cells (1×10^6 cells/well) were transfected with control or miR-4669 mimic (20 nM) for 24 hours and exosomes were purified from the culture supernatant (10 ml) by using an ExoQuick-TC (System Biosciences, Mountain View, CA, USA) followed by extraction of exosomal RNA for quantitative real-time PCR analysis. **, $P < 0.01$.
